# Author Correction: Nrf2 contributes to the weight gain of mice during space travel

**DOI:** 10.1038/s42003-020-01292-7

**Published:** 2020-10-07

**Authors:** Takafumi Suzuki, Akira Uruno, Akane Yumoto, Keiko Taguchi, Mikiko Suzuki, Nobuhiko Harada, Rie Ryoke, Eriko Naganuma, Nanae Osanai, Aya Goto, Hiromi Suda, Ryan Browne, Akihito Otsuki, Fumiki Katsuoka, Michael Zorzi, Takahiro Yamazaki, Daisuke Saigusa, Seizo Koshiba, Takashi Nakamura, Satoshi Fukumoto, Hironobu Ikehata, Keizo Nishikawa, Norio Suzuki, Ikuo Hirano, Ritsuko Shimizu, Tetsuya Oishi, Hozumi Motohashi, Hirona Tsubouchi, Risa Okada, Takashi Kudo, Michihiko Shimomura, Thomas W. Kensler, Hiroyasu Mizuno, Masaki Shirakawa, Satoru Takahashi, Dai Shiba, Masayuki Yamamoto

**Affiliations:** 1 Department of Medical Biochemistry, Tohoku University Graduate School of Medicine, Sendai, Japan; 2 Department of Integrative Genomics, Tohoku Medical Megabank Organization, Tohoku University, Sendai, Japan; 3 JEM Utilization Center, Human Spaceflight Technology Directorate, JAXA, Tsukuba, Japan; 4 The Advanced Research Center for Innovations in Next-Generation Medicine (INGEM), Tohoku University, Sendai, Japan; 5 Center for Radioisotope Sciences, Tohoku University Graduate School of Medicine, Sendai, Japan; 6 Institute for Animal Experimentation, Tohoku University Graduate School of Medicine, Sendai, Japan; 7 Department of Functional Brain Imaging, Institute of Development, Aging, and Cancer, Tohoku University, Sendai, Japan; 8 Department of Molecular Hematology, Tohoku University Graduate School of Medicine, Sendai, Japan; 9 Department of Molecular Biotechnology and Health Sciences, University of Turin, 10125 Torino, Italy; 10 Division of Molecular Pharmacology & Cell Biophysics, Tohoku University Graduate School of Dentistry, Sendai, Japan; 11 Division of Pediatric Dentistry, Tohoku University Graduate School of Dentistry, Sendai, Japan; 12 Immunology Frontier Research Center, Osaka University, Suita, Japan; 13 Division of Oxygen Biology, Tohoku University Graduate School of Medicine, Sendai, Japan; 14 Department of Gene Expression Regulation, Institute of Development, Aging and Cancer, Tohoku University, Sendai, Japan; 15 Laboratory Animal Resource Center in Transborder Medical Research Center, and Department of Anatomy and Embryology, Faculty of Medicine, University of Tsukuba, Tsukuba, Japan; 16 Translational Research Program, Fred Hutchinson Cancer Research Center, Seattle, WA USA

**Keywords:** Experimental models of disease, Mouse

Correction to: *Communications Biology* 10.1038/s42003-020-01227-2, published online 08 September 2020.

In the originally published version of the Article, an incorrect file was uploaded as Supplementary Movie 1. The error has been corrected in the HTML version of the Article.

