# Supplementary material for: Nrf2 contributes to the weight gain of mice during space travel
Source: Commun Biol. 2020 Sep 8;3:496. doi: 10.1038/s42003-020-01227-2 (PMC7479603; doi:10.1038/s42003-020-01227-2)
Supplement: Supplementary file 1 — Supplementary Information [file 42003_2020_1227_MOESM1_ESM.pdf]

Supplementary Figure 1

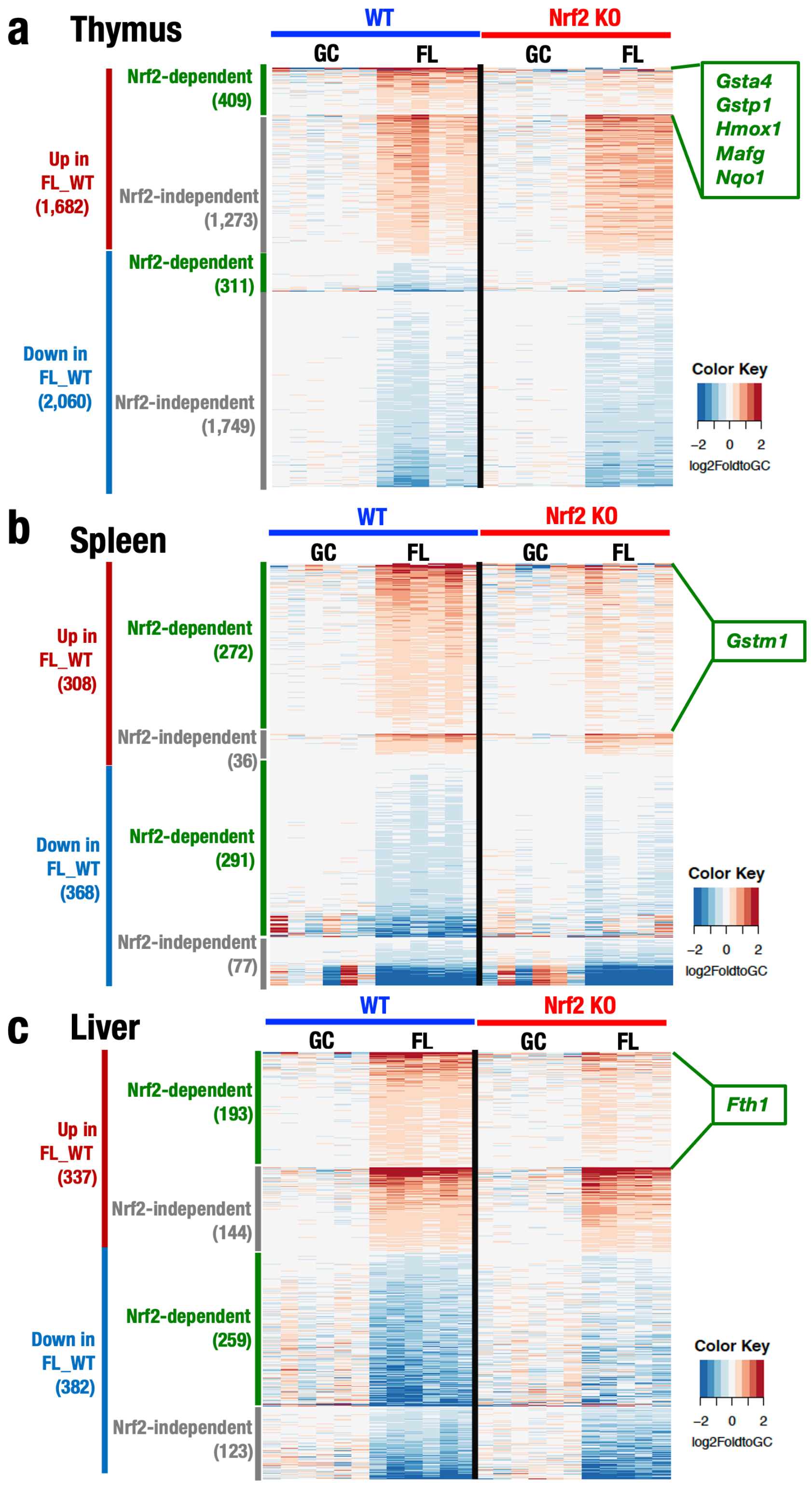

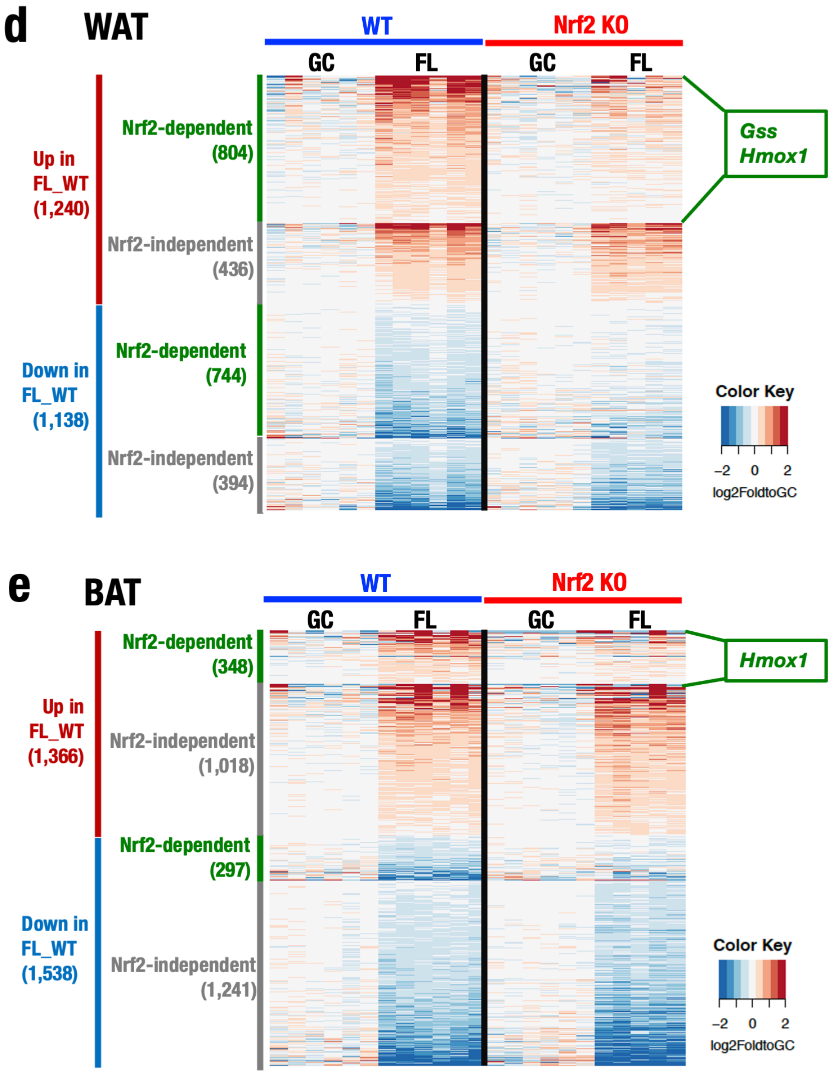

Supplementary Figure 1. Classification of space-induced changes of gene expression ($p<0.05$) into Nrf2-dependent group and Nrf2-independent group by comparing GC Nrf2KO with FL Nrf2-KO in thymus (a), spleen (b), liver (c), WAT (d) and BAT (e). Values of GC WT and GC Nrf2KO are set as 0. n=6 for GC WT, FL WT and GC KO, and n=5 for FL KO.

Supplementary Figure 2

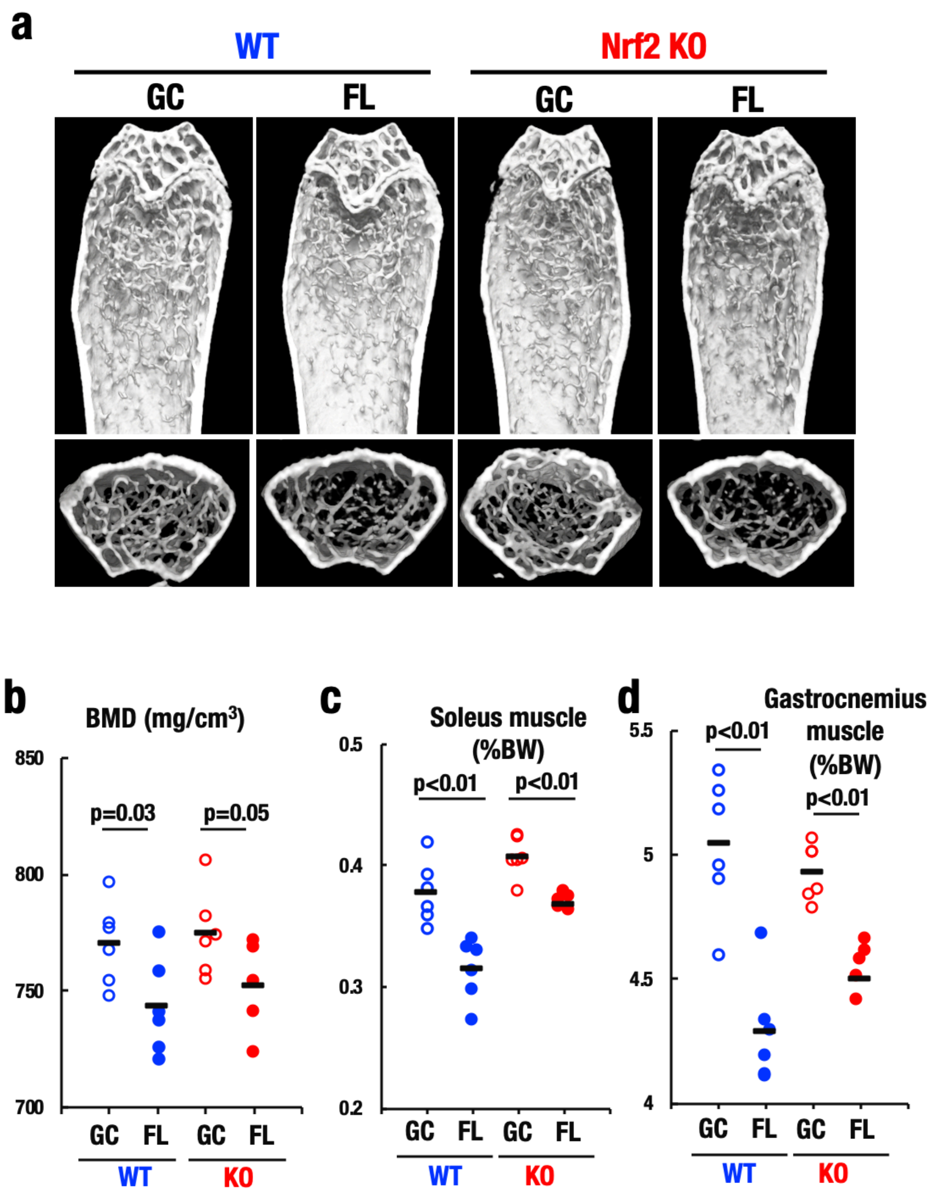

Supplementary Figure 2. (**a**) Representative microCT images of the femur of WT and Nrf2-KO mice in GC and FL groups (Upper: longitudinal view; Lower: axial view of the metaphyseal region). (**b**) Bone mineral density (BMD) of the femurs of WT and Nrf2-KO mice in GC and FL groups. (**c,d**) Body weight-normalized masses of soleus muscle (**c**) and gastrocnemius muscle (**d**). Data are presented as mean, and dots represent individual animals. n=6 for GC WT, FL WT and GC KO, and n=5 for FL KO. One-way ANOVA with Tukey-Kramer test.

Supplementary Figure 3

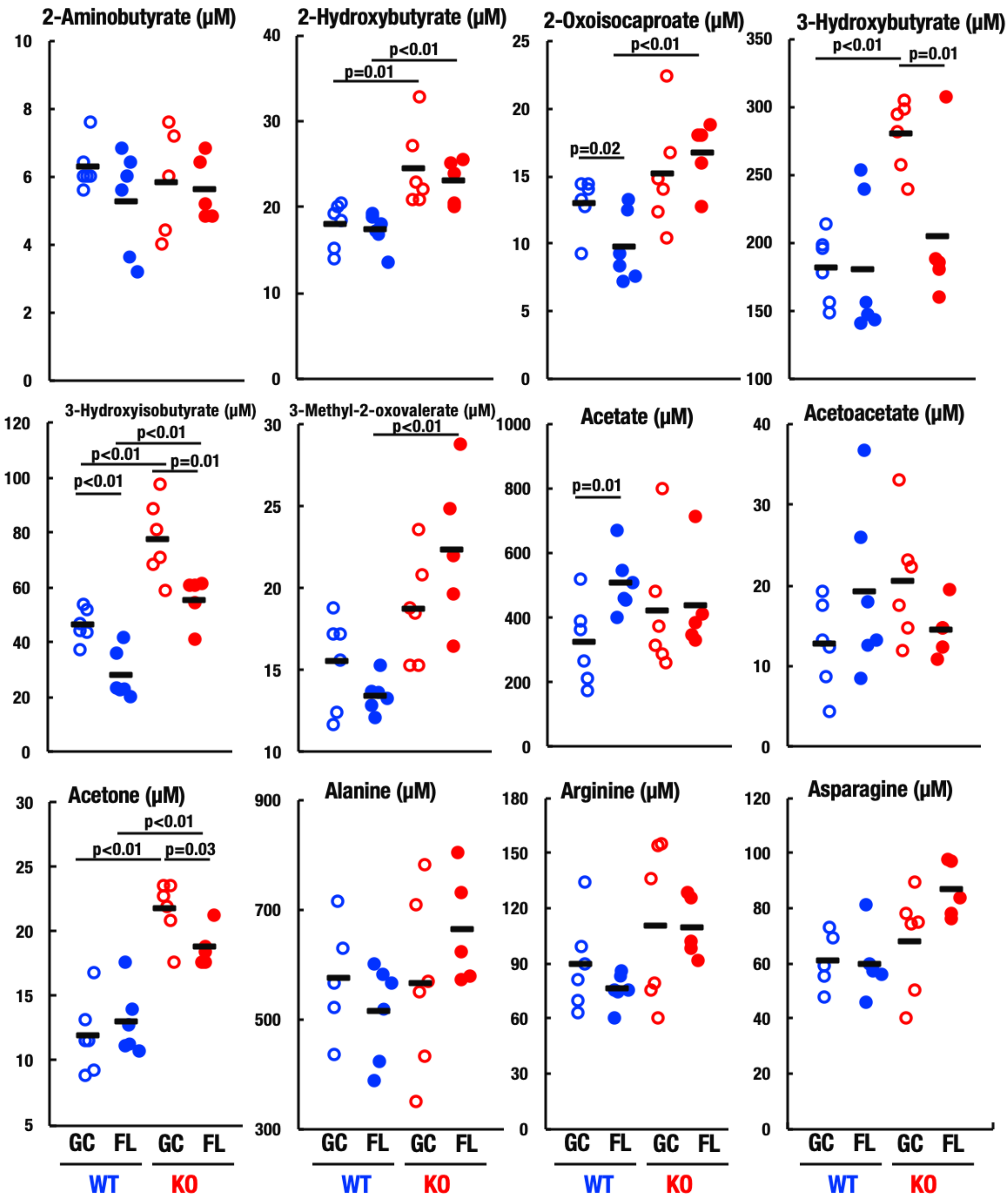

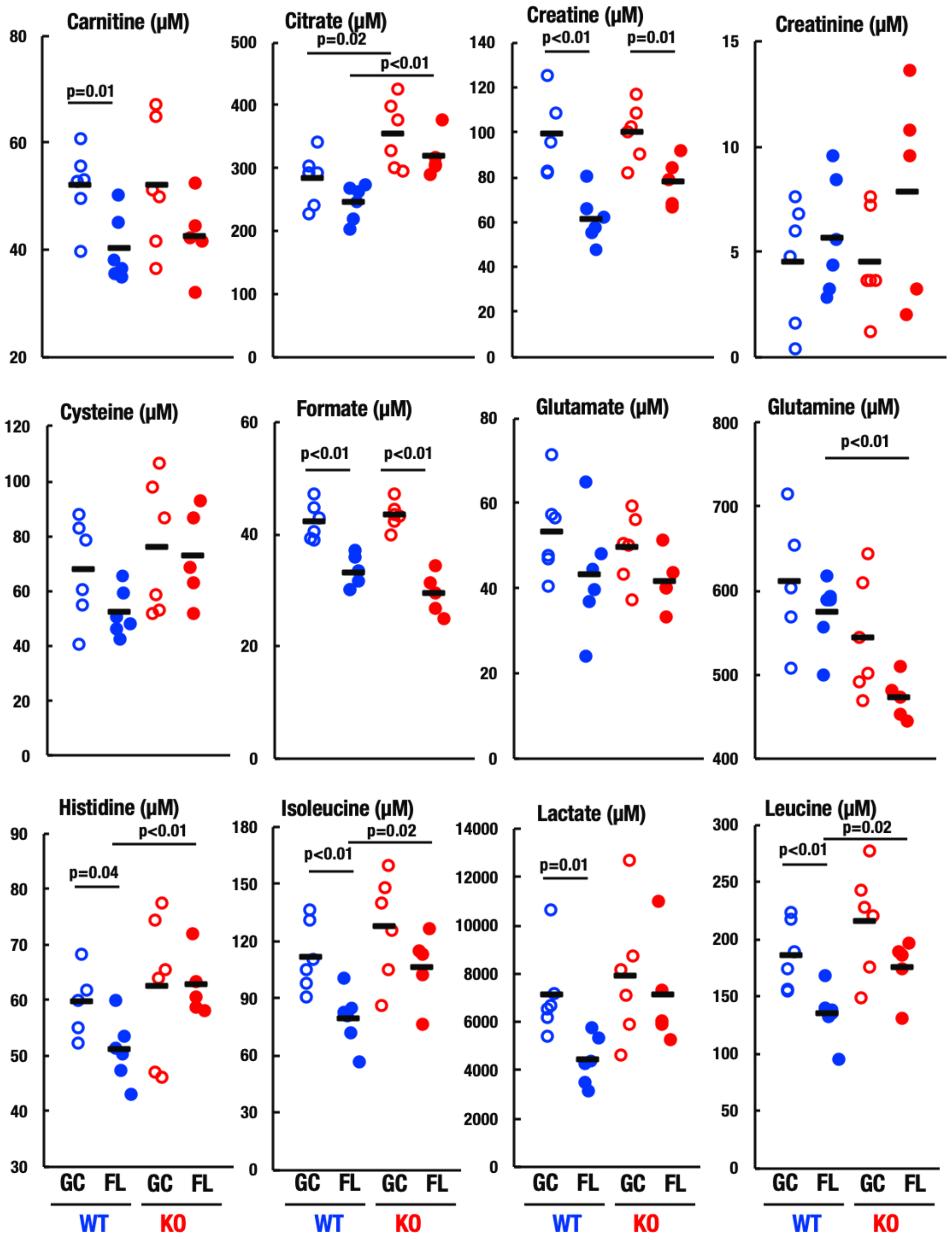
Carnitine (μM)
p=0.01
Citrate (μM)
p=0.02
p<0.01
Creatine (μM)
p<0.01
p=0.01
Creatinine (μM)
Cysteine (μM)
Formate (μM)
p<0.01
p<0.01
Glutamate (μM)
Glutamine (μM)
p<0.01
Histidine (μM)
p<0.01
p=0.04
Isoleucine (μM)
p=0.02
p<0.01
Lactate (μM)
p=0.01
Leucine (μM)
p=0.02
p<0.01
GC FL GC FL
WT KO

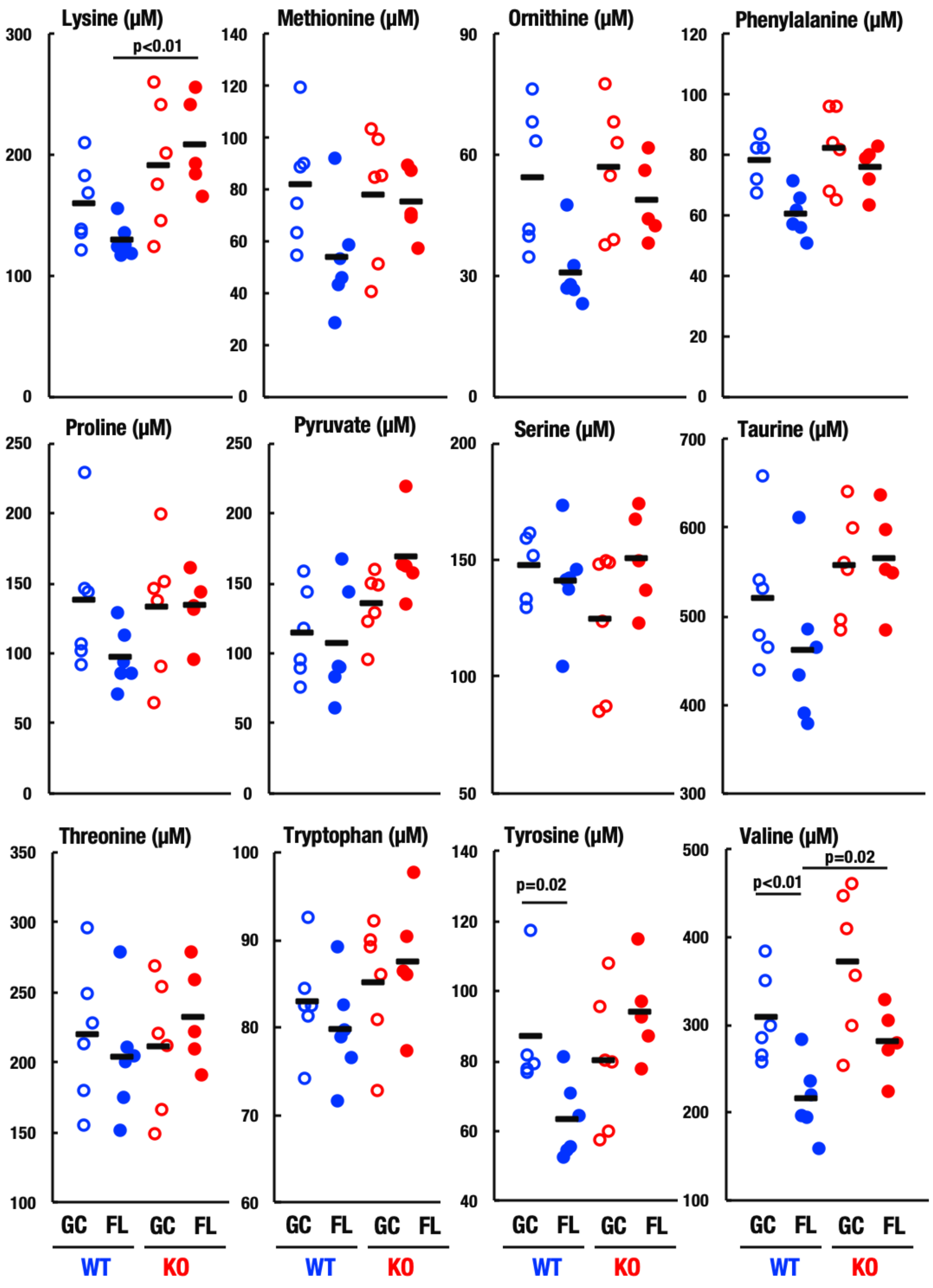

Supplementary Figure 3. Levels of plasma metabolites examined by NMR-based metabolome analyses. Data are presented as mean, and dots represent individual animals. n=6 for GC WT, FL WT and GC KO, and n=5 for FL KO. One-way ANOVA with Tukey-Kramer test.

Supplementary Figure 4

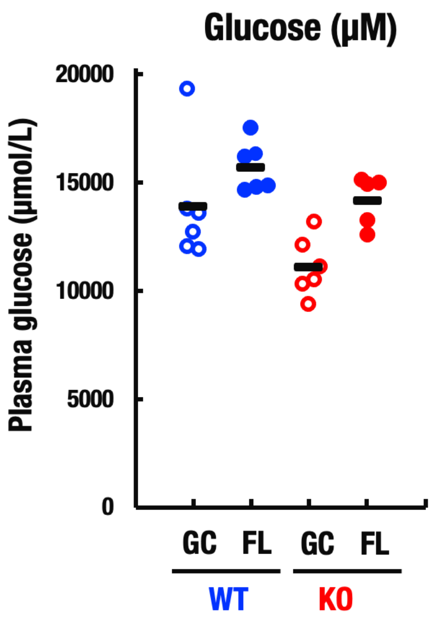

Supplementary Figure 4. Levels of plasma glucose from WT and Nrf2-KO mice in GC and FL groups. Data are presented as mean, and dots represent individual animals. n=6 for GC WT, FL WT and GC KO, and n=5 for FL KO.

Supplementary Figure 5

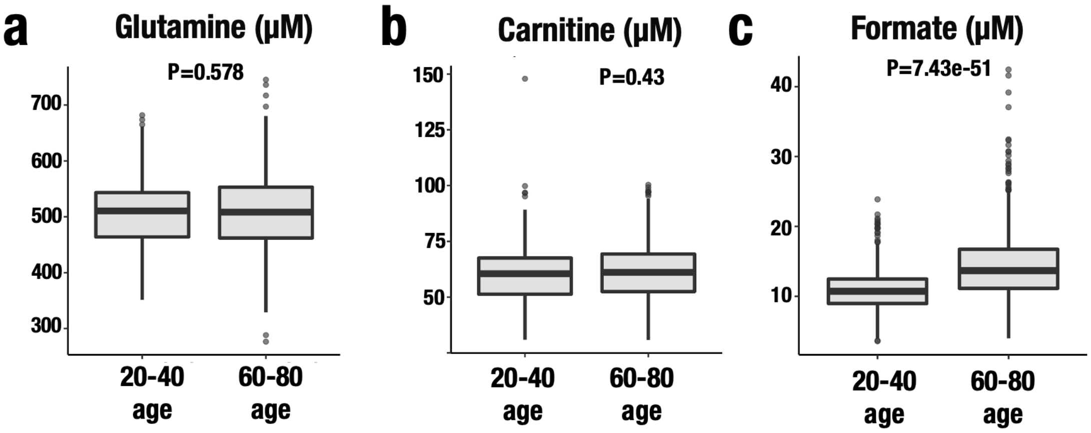

Supplementary Figure 5. Age-distribution of plasma levels of glutamine (**a**), carnitine (**b**) and formate (**c**) in a human cohort of the ToMMo study (data replotted from ref. 22). P values were calculated with Wilcoxon-Mann-Whitney test. n = 545 (20-40 age) and 955 (60-80 age) for **a**. n = 472 (20-40 age) and 830 (60-80 age) for **b**. n = 529 (20-40 age) and 955 (60-80 age) for **c**.

Supplementary Figure 6

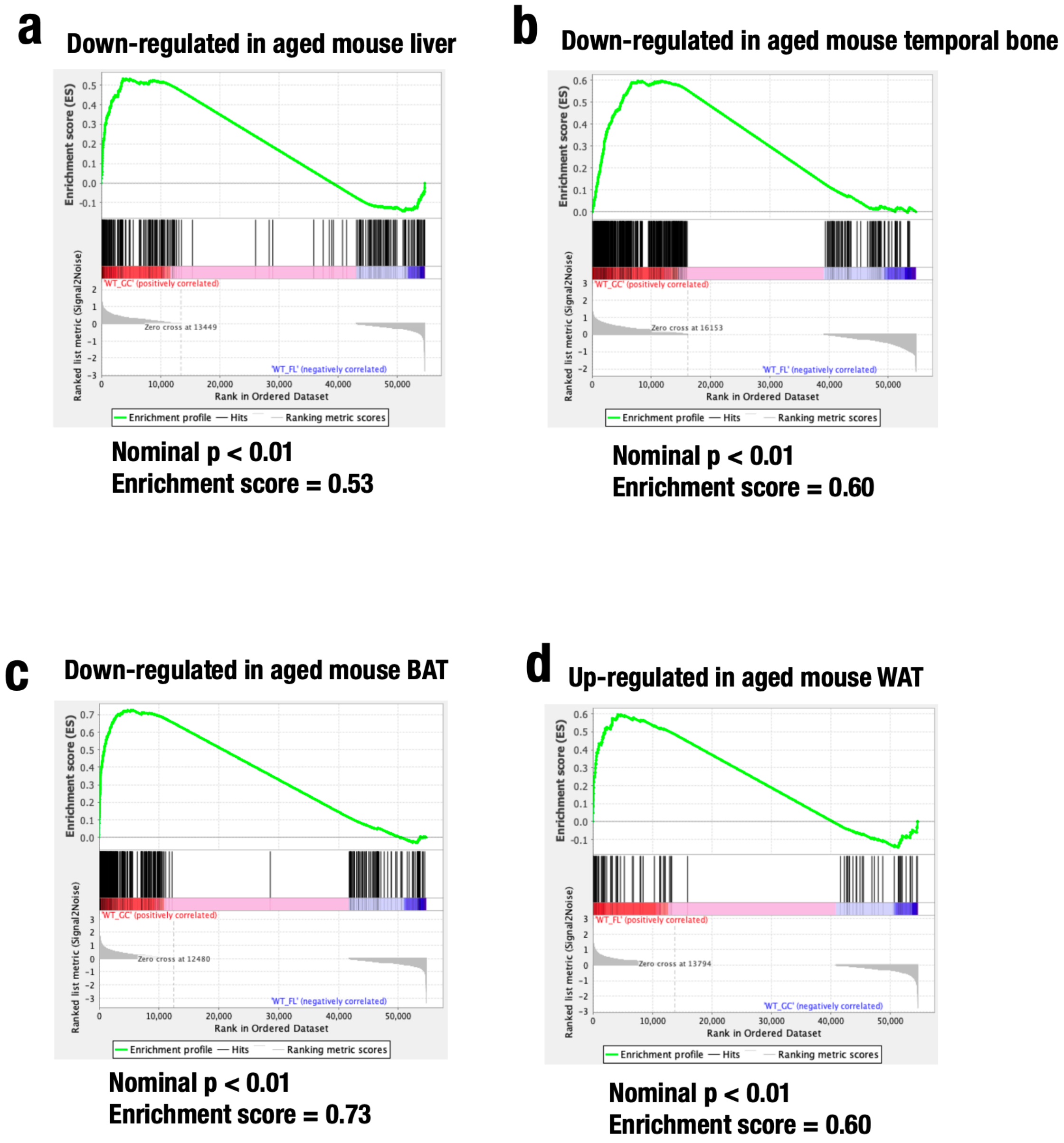

Supplementary Figure 6. Gene set enrichment analyses (GSEA) of gene expression induced by space flight in liver (a), temporal bone (b), BAT (c) and WAT (d).

Supplementary Figure 7

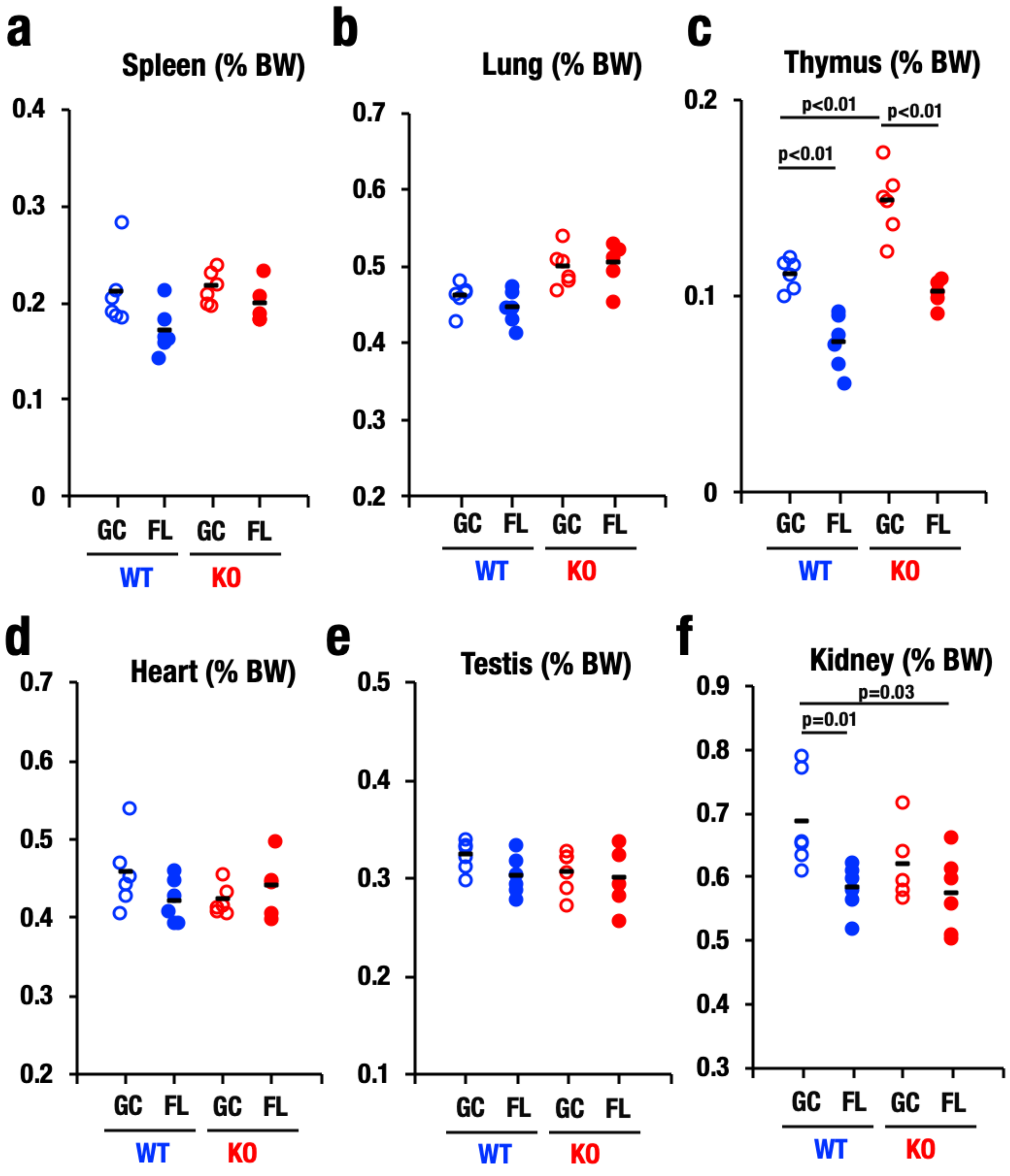

Supplementary Figure 7. (**a-f**) Body weight-normalized masses of spleen (**a**), lung (**b**), thymus (**c**), heart (**d**), testis (**e**), and kidney (**f**) in GC and FL groups. Data are presented as mean, and dots represent individual animals. n=6 for GC WT, FL WT and GC KO, and n=5 for FL KO. One-way ANOVA with Tukey-Kramer test.

Supplementary Figure 8

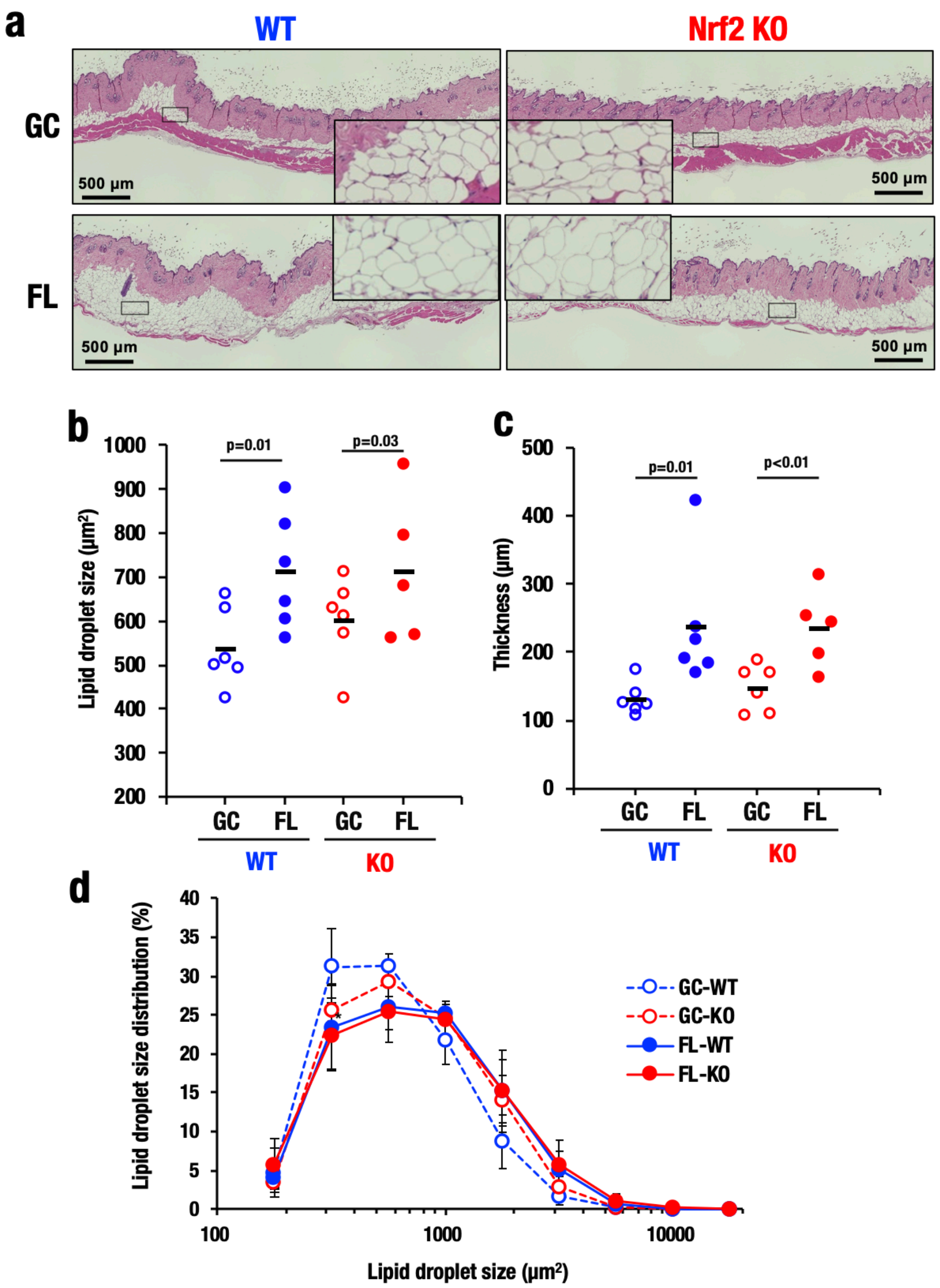

Supplementary Figure 8. (**a**) Histological images of skin from WT and Nrf2-KO mice in GC and FL groups. (**b**) Lipid droplet size of subcutaneous fat from WT and Nrf2-KO mice in GC and FL groups. (**c**) Thickness of subcutaneous fat from WT and Nrf2-KO mice in GC and FL groups. (**d**) Lipid droplet size distribution of subcutaneous fat from WT and Nrf2-KO mice in GC and FL groups. Data are presented as mean. Dots represent individual animals for **b** and **c**. n=6 for GC WT, FL WT and GC KO, and n=5 for FL KO. One-way ANOVA with Tukey-Kramer test.

Supplementary Figure 9

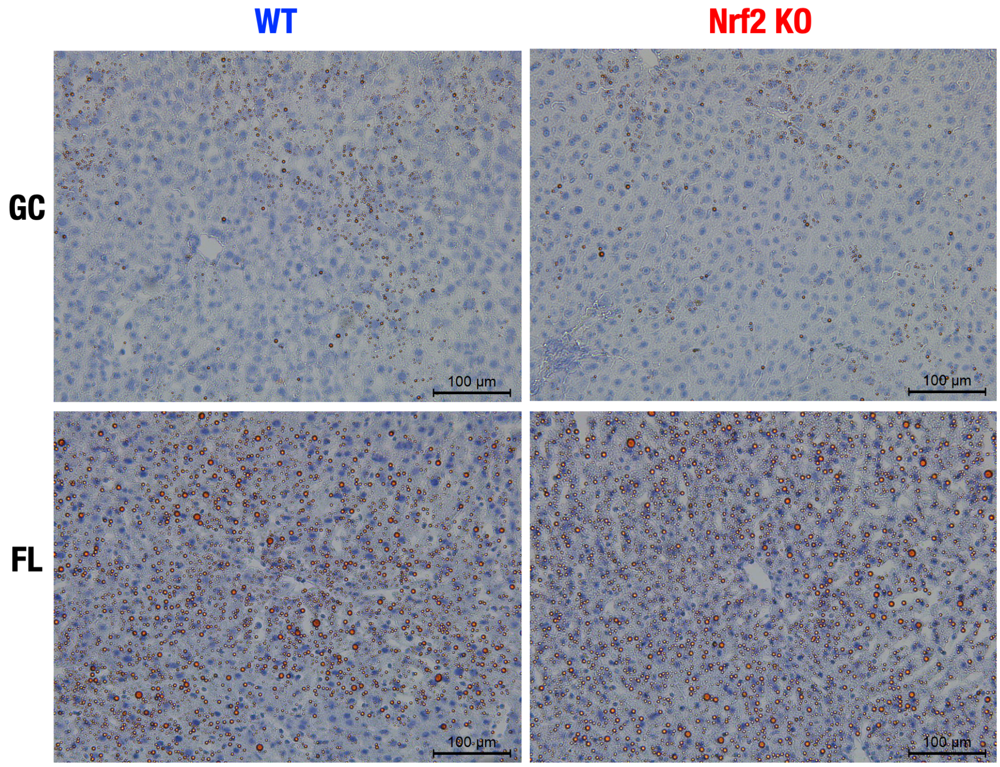

Supplementary Figure 9. Oil Red O staining images of liver from WT and Nrf2-KO mice in GC and FL groups.

Supplementary Figure 10

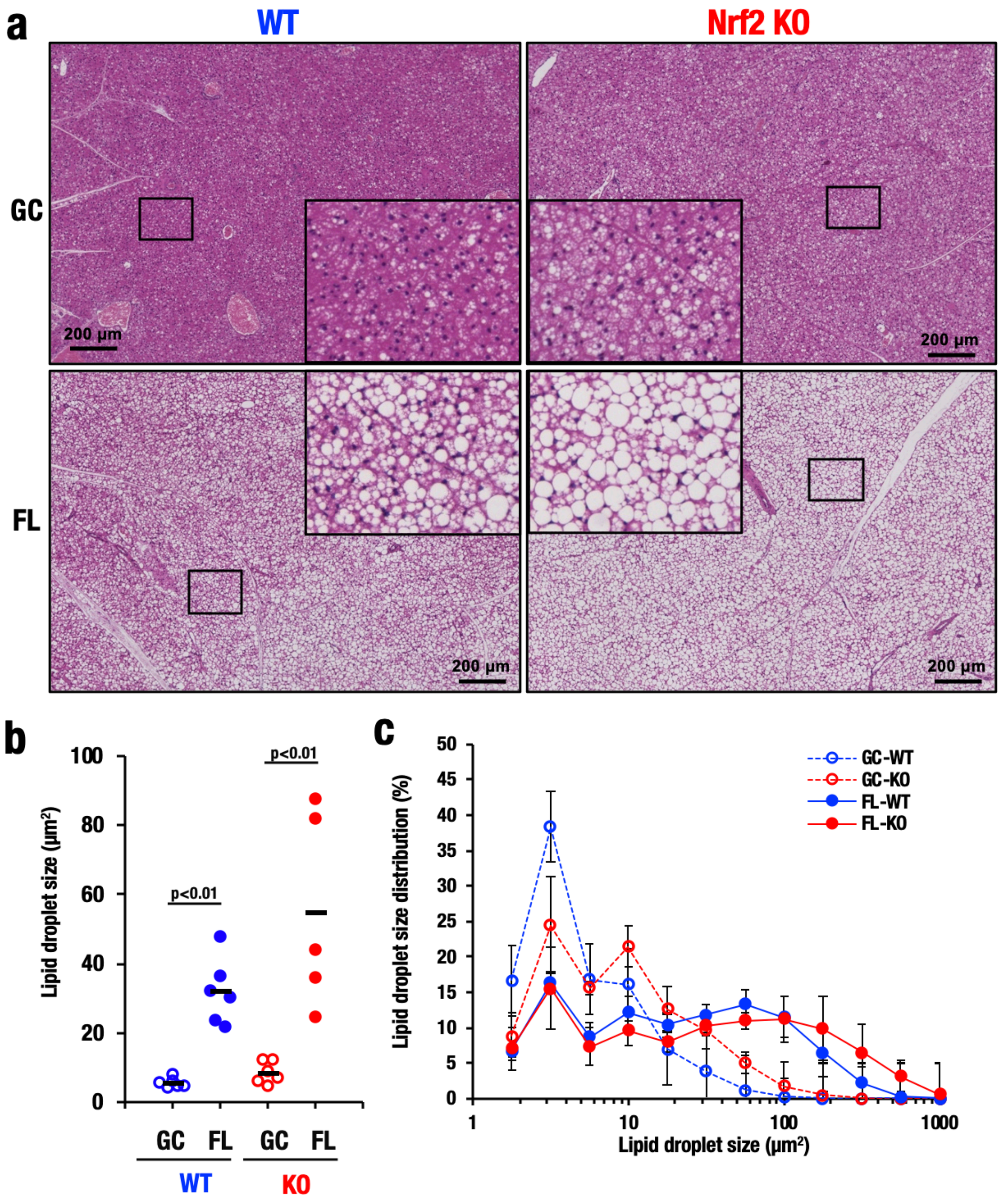

Supplementary Figure 10. (**a**) Histological images of iBAT from WT and Nrf2-KO mice in GC and FL groups. (**b**) Lipid droplet size of iBAT from WT and Nrf2-KO mice in GC and FL groups. Data are presented as mean, and dots represent individual animals. n=6 for GC WT, FL WT and GC KO, and n=5 for FL KO. One-way ANOVA with Tukey-Kramer test. (**c**) Lipid droplet size distribution of iBAT from WT and Nrf2-KO mice in GC and FL groups.

Supplementary Figure 11

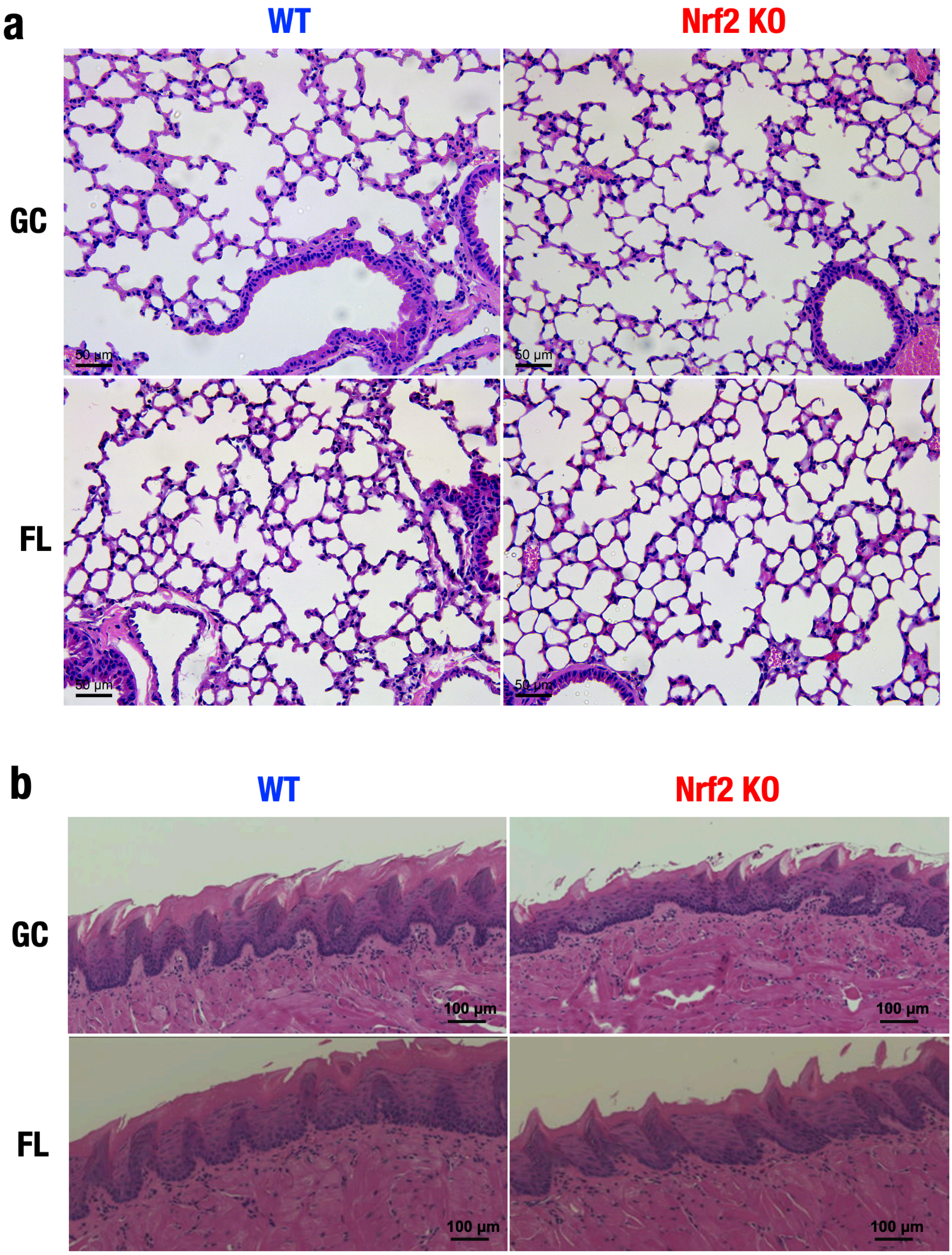

Supplementary Figure 11. Histological images of lung (**a**) and tongue (**b**) from WT and Nrf2-KO mice in GC and FL groups.

Supplementary Figure 12

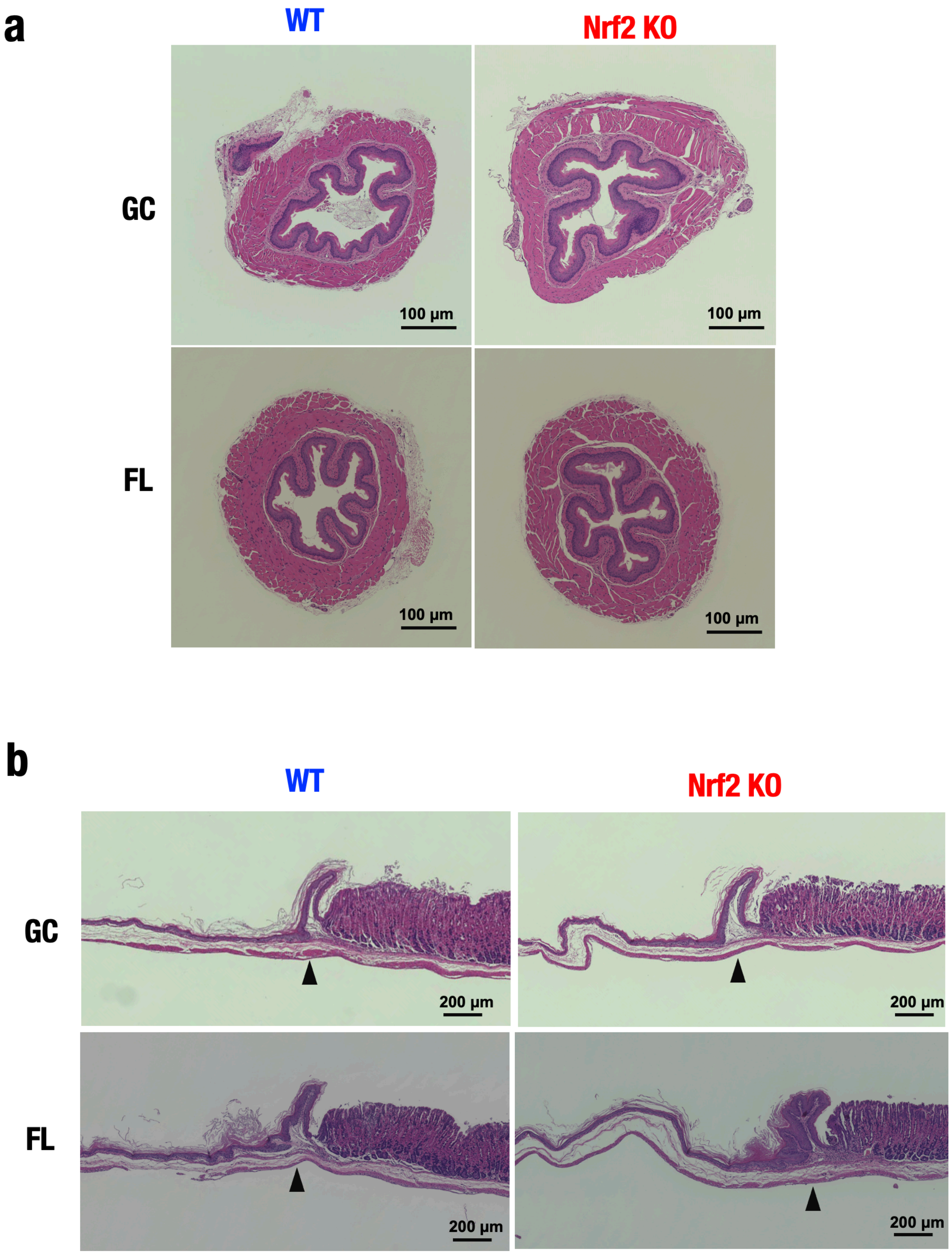

Supplementary Figure 12. Histological images of esophagus (**a**) and stomach (**b**) from WT and Nrf2-KO mice in GC and FL groups.

Supplementary Figure 13

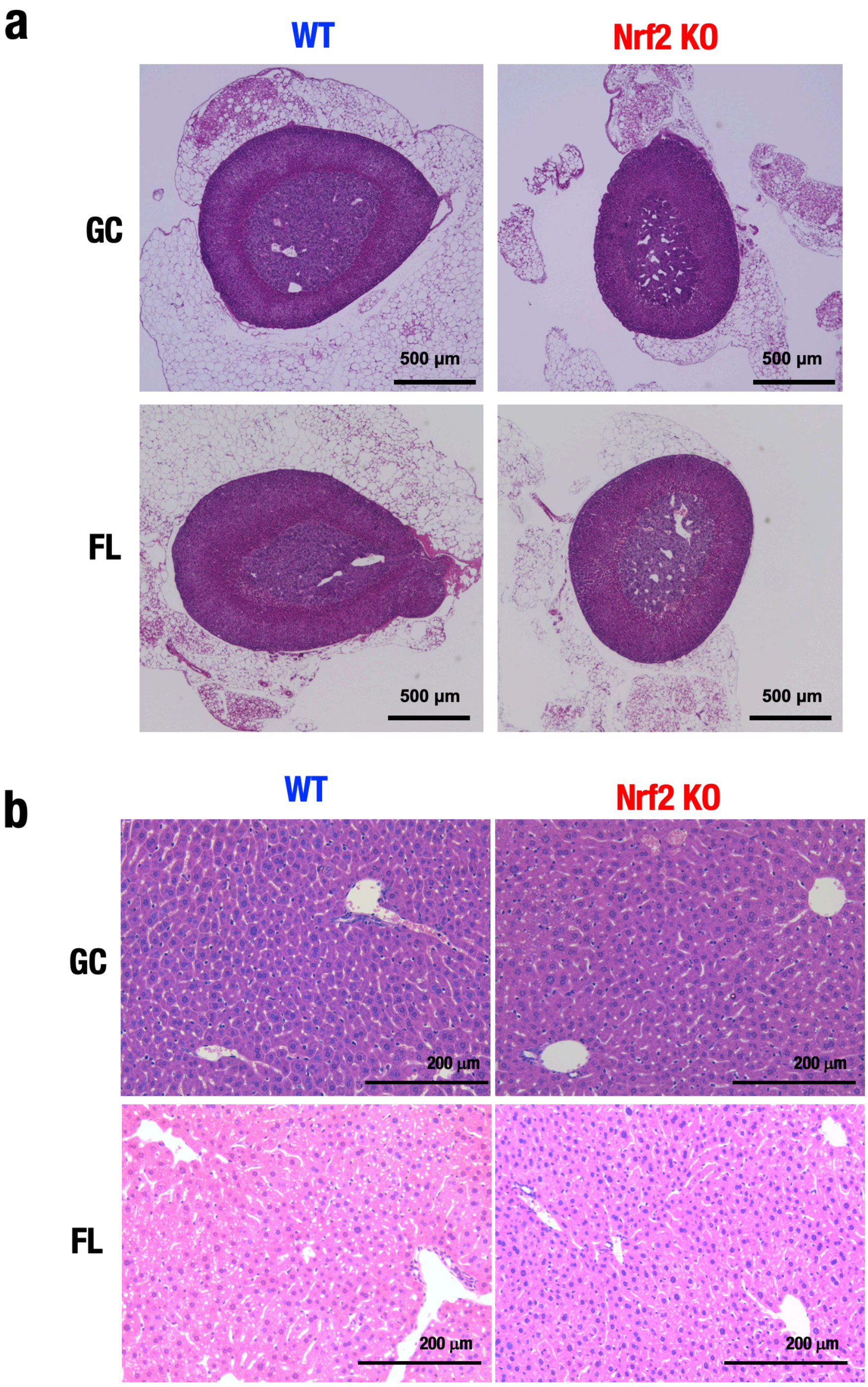

Supplementary Figure 13. Histological images of adrenal gland (**a**) and liver (**b**) from WT and Nrf2-KO mice in GC and FL groups.

Supplementary Figure 14

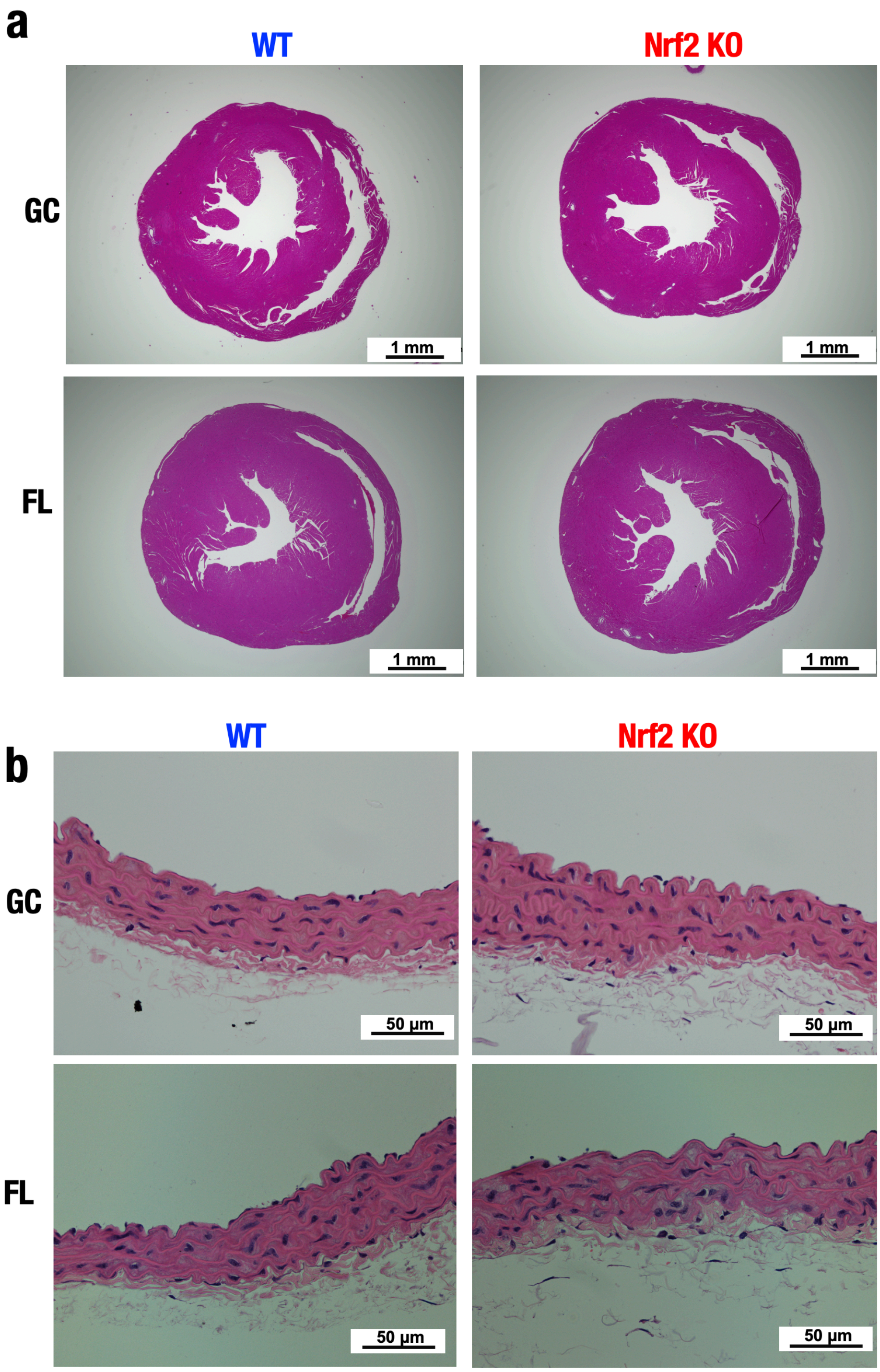

Supplementary Figure 14. Histological images of heart (**a**) and aorta (**b**) from WT and Nrf2-KO mice in GC and FL groups.
