# Supplementary material for: Nrf2 contributes to the weight gain of mice during space travel
Source: Commun Biol. 2020 Sep 8;3:496. doi: 10.1038/s42003-020-01227-2 (PMC7479603; doi:10.1038/s42003-020-01227-2)
Supplement: Supplementary file 2 — Description of Additional Supplementary Files [file 42003_2020_1227_MOESM2_ESM.pdf]

Descriptions of Additional Supplementary Files

Supplementary movie 1: A video of WT and Nrf2-KO mice onboard the ISS in the HCU.

Supplementary movie 2: A video of representative appearance of Nrf2-KO (M6 and A3) and WT (M4 and A4) mice after flight return (M6 and M4) and end of experiment ground control mice (A3 and A4).

Supplementary data 1: Gene set enrichment analysis (GSEA) of gene expression induced by space flight in WT mice.

Supplementary data 2: All source data for graphs and charts presented in the main figures.
