# Supplementary material for: Nrf2 contributes to the weight gain of mice during space travel
Source: Commun Biol. 2020 Sep 8;3:496. doi: 10.1038/s42003-020-01227-2 (PMC7479603; doi:10.1038/s42003-020-01227-2)
Supplement: Supplementary file 7 — Reporting Summary [file 42003_2020_1227_MOESM7_ESM.pdf]

nature research

Corresponding author(s): Masayuki Yamamoto

Last updated by author(s): Jul 26, 2020

Reporting Summary

Nature Research wishes to improve the reproducibility of the work that we publish. This form provides structure for consistency and transparency in reporting. For further information on Nature Research policies, see Authors & Referees and the Editorial Policy Checklist .

Statistics

For all statistical analyses, confirm that the following items are present in the figure legend, table legend, main text, or Methods section.

n/a	Confirmed	
☐	☒	The exact sample size (*n*) for each experimental group/condition, given as a discrete number and unit of measurement
☐	☒	A statement on whether measurements were taken from distinct samples or whether the same sample was measured repeatedly
☐	☒	The statistical test(s) used AND whether they are one- or two-sided<br>*Only common tests should be described solely by name; describe more complex techniques in the Methods section.*
☒	☐	A description of all covariates tested
☒	☐	A description of any assumptions or corrections, such as tests of normality and adjustment for multiple comparisons
☐	☒	A full description of the statistical parameters including central tendency (e.g. means) or other basic estimates (e.g. regression coefficient) AND variation (e.g. standard deviation) or associated estimates of uncertainty (e.g. confidence intervals)
☐	☒	For null hypothesis testing, the test statistic (e.g. *F*, *t*, *r*) with confidence intervals, effect sizes, degrees of freedom and *P* value noted<br>*Give P values as exact values whenever suitable.*
☒	☐	For Bayesian analysis, information on the choice of priors and Markov chain Monte Carlo settings
☒	☐	For hierarchical and complex designs, identification of the appropriate level for tests and full reporting of outcomes
☒	☐	Estimates of effect sizes (e.g. Cohen's *d*, Pearson's *r*), indicating how they were calculated

Our web collection on statistics for biologists contains articles on many of the points above.

Software and code

Policy information about availability of computer code

Data collection	ScanXmate-A100S Scanner for MicroCT scanning, HiSeq2500 for sequencing, Bruker Avance 600-MHz spectrometer or UltiMateTM 3000 RSLC system and FTMS for plasma analysis,
Data analysis	TRI/3D-BON software for reconstruction of three-dimensional micro-structural image and calculation of bone mineral density, STAR (version 2.6.1) for mapping to mouse mm10 genome, RSEM (version 1.3.1) for measurement of gene expression, Subio Platform software for normalization of TPM, R software (version 3.5.1; www.r-project.org) for PCA analysis, R-based heatmap.2 for generating heatmap, DESeq2 (version 1.22.2) for differential gene expression analysis, GSEA (www.broad.mit.edu/gsea) for gene set enrichment analysis, BZ-X800 (Keyence) and ImageJ for measurement of lipid droplet, Graphpad Prism, Excel and JMP Pro for statistic analysis

For manuscripts utilizing custom algorithms or software that are central to the research but not yet described in published literature, software must be made available to editors/reviewers. We strongly encourage code deposition in a community repository (e.g. GitHub). See the Nature Research guidelines for submitting code & software for further information.

Data

Policy information about availability of data

All manuscripts must include a data availability statement. This statement should provide the following information, where applicable:

- Accession codes, unique identifiers, or web links for publicly available datasets
- A list of figures that have associated raw data
- A description of any restrictions on data availability

The data discussed in this publication have been deposited in NCBI's Gene Expression Omnibus and are accessible through GEO Series accession number GSE152382 (https://www.ncbi.nlm.nih.gov/geo/query/acc.cgi?acc=GSE152382). All relevant data are available from the corresponding authors upon reasonable request.

Field-specific reporting

Please select the one below that is the best fit for your research. If you are not sure, read the appropriate sections before making your selection.

[x] Life sciences   [ ] Behavioural & social sciences   [ ] Ecological, evolutionary & environmental sciences

For a reference copy of the document with all sections, see nature.com/documents/nr-reporting-summary-flat.pdf

Life sciences study design

All studies must disclose on these points even when the disclosure is negative.

Sample size	A mouse space experiment: 6 in each group.
Data exclusions	Due to flight accident, data from flight Nrf2KO No.4 mouse were deemed outliers and excluded.
Replication	All experiments included contain independent biological replicates.
Randomization	Data were not analyzed with randomization.
Blinding	Investigators were not blinded during experiment.

Reporting for specific materials, systems and methods

We require information from authors about some types of materials, experimental systems and methods used in many studies. Here, indicate whether each material, system or method listed is relevant to your study. If you are not sure if a list item applies to your research, read the appropriate section before selecting a response.

Materials & experimental systems

n/a	Involved in the study	
[x]	[ ]	Antibodies
[x]	[ ]	Eukaryotic cell lines
[x]	[ ]	Palaeontology
[ ]	[x]	Animals and other organisms
[x]	[ ]	Human research participants
[x]	[ ]	Clinical data

Methods

n/a	Involved in the study	
[x]	[ ]	ChIP-seq
[x]	[ ]	Flow cytometry
[x]	[ ]	MRI-based neuroimaging

Animals and other organisms

Policy information about studies involving animals; ARRIVE guidelines recommended for reporting animal research

Laboratory animals	Nrf2-KO (Nfe212tm1Ymk) and age-matched WT male mice in the C57BL/6J background were used
Wild animals	No wild animals was used in this study.
Field-collected samples	This study does not involve samples collected from the field.
Ethics oversight	All animal experiments were approved by the Institutional Animal Care and Use Committees of JAXA (protocol numbers 017-001 and 017-014), NASA (protocol number FLT-17-112), and Explora BioLabs (EB15-010C), and conducted according to the related guidelines and applicable laws of Japan and the United States of America.

Note that full information on the approval of the study protocol must also be provided in the manuscript.
